# Localized chromophobe renal cell carcinoma: preoperative imaging judgment and laparoscopic simple enucleation for treatment

**DOI:** 10.1590/S1677-5538.IBJU.2017.0519

**Published:** 2018

**Authors:** Wenbiao Ren, Bichen Xue, Jiandong Qu, Longfei Liu, Chao Li, Xiongbing Zu

**Affiliations:** 1 Department of Urology, Xiangya Hospital, Central South University, Changsha, China

**Keywords:** Carcinoma, Renal Cell, Laparoscopy, Therapeutics

## Abstract

**Objective:**

To evaluate the preoperative imaging manifestation and therapeutic effect of laparoscopic simple enucleation (SE) for localized chromophobe renal cell carcinoma (chRCC).

**Materials and Methods:**

Clinical data of 36 patients who underwent laparoscopic SE of localized chRCC at our institute were retrospectively analyzed. All patients underwent preoperative renal protocol CT (unenhanced, arterial, venous, and delayed images). CT scan characteristics were evaluated. After intraoperative occlusion of the renal artery, the tumor was free bluntly along the pseudocapsule and enucleated totally. The patients were followed up regularly after the operation.

**Results:**

Mean tumor diameter was 3.9±1.0 cm, 80% of tumors were homogeneous and all the tumors had complete pseudocapsule. The attenuation values were slightly lower than normal renal cortex and degree of enhancement of the tumors were significantly lower than normal renal cortex. Mean operation time was 104.3±18.2 min. Mean warm ischemia time (WIT) was 21.3±3.5 min. Mean blood loss was 78.6±25.4 mL. No positive surgical margin was identified. Mean postoperative hospital stay was 5.3±1.5 d. Hematuria occurred in 3 patients and all disappeared within 3 days. After a mean follow-up of 32.1±20.6 months, no patient had local recurrence or metastatic progression.

**Conclusion:**

Localized chRCCs have a great propensity for homogeneity and complete pseudocapsule. The attenuation values were slightly lower than normal renal cortex and small degree of enhancement. Laparoscopic SE is a safe and effective treatment for localized chRCC. The oncological results were satisfactory.

## INTRODUCTION

Renal cell carcinoma is a common malignant tumor in the urinary system. Among the different sub-types of renal cell carcinoma, chromophobe renal cell carcinoma (chRCC) accounts for approximately 3% to 5% of all RCCs (1). ChRCC is considered to be derived from the collecting duct, harbors mitochondria alterations, and can be observed in Birt-Hogg-Dubé syndrome (2). In general, chRCCs have been considered to be homogeneous and indolent tumors. It has been evidenced that the degree of early enhancement during the corticomedullary phase was largest for clear cell RCC followed by oncocytoma, chRCC, and papillary RCC (3). However, as far as it is known, few studies are specialized in the imaging manifestations of chRCCs. Among all the different RCC subtypes, chRCCs reportedly have the best prognosis, with a 5-year survival rate of over 90%, as opposed to clear cell and papillary RCCs, which have rates of survival of 55% - 60% and 80% −90%, respectively (4, 5).

The treatments of localized chRCC include active surveillance, radiofrequency ablation, radical nephrectomy and nephron-sparing surgery. Simple enucleation (SE) is a nephron sparing surgery, which dissects the tumor bluntly along the natural cleavage plane between the tumor pseudocapsule and healthy kidney parenchyma (6). SE was first used in benign renal tumors. Nowadays, SE is routinely performed for familial RCC and sporadic RCC, which can retain the maximum normal parenchyma in order to reduce the risk of chronic kidney disease development (7, 8). Recently, it has been reported that tumor SE had revealed comparable oncologic outcomes compared with standard margin partial nephrectomy for RCC (9–12). However, it is understood that, most of the previous articles were about SE in the treatment of clear cell RCCs. No previous studies have evaluated safety and feasibility of this technique in chRCCs exclusively. Ficarra V, et al. (13) analyzed a lot of previous data and conclude that simple enucleation was a surgical technique responding to the EAU guidelines criteria for oncologic safety. First laparoscopic SE for chRCC was performed in November 2010 at our institute.

Therefore, the purpose of this study is to evaluate the CT manifestations, enhancement features and the safety, feasibility and oncological outcomes treatment with laparoscopic SE in a series of pathologically proven localized chRCCs at our institute.

## MATERIALS AND METHODS

### 

#### Patients

This was a single center, institutional review board approved, retrospective study. Consecutive patients were selected fulfilling the following criteria:

1. Preoperative protocol CT (unenhanced, arterial, venous, and delayed images) of the urinary system was performed in the hospital within 1 month from the day of surgery.
2. Postoperative pathology were chRCCs reconfirmed by uropathologists according to the 2016 WHO classification of renal tumors (14).
3. Treatment was laparoscopic SE surgery performed by experienced urological doctors.

Patients with doubtful tumor histology, distant metastasis or irregular follow-up were excluded. Patients treated with normal margin of partial nephrectomy were also excluded.

#### Preoperative evaluation

Preoperative evaluation was conducted routinely and consisted of the following: chief complaint, physical examination, routine blood test, chest radiograph, electrocardiogram and protocol CT (unenhanced, arterial, venous, and delayed images) of the urinary system. The complexity of tumors was measured by PADUA score system (15).

The maximum diameter, texture and capsule of the tumor were observed. The presence of distant metastatic disease, suspicious lymphadenopathy, or tumor thrombus was noted. The average attenuation values of tumor and normal renal cortex at 4 various stages were recorded.

### Surgical technique

Generally anesthetized patients were placed in lateral decubitus position. Three trocars were placed on the affected flank through retroperitoneal laparoscopic approach. The renal artery was fully dissected and blocked with an artery clamp. The renal parenchyma was incised sharply through a 5 mm length, adjacent to the tumor fringe. When the proper surgical plane is entered and the capsule is reached, the tumor can be easily isolated bluntly from the normal acroteric parenchyma and simply enucleated from the kidney without any visible rim of normal parenchyma. A single-layer suture with Hem-lock placed on the renal capsule at the beginning and end of the suture process was applied. Then, the artery clamp was taken off from the renal pedicle. The operation time, warm ischemia time (WIT) and blood loss were recorded.

#### Postoperative management

Surgical specimens were processed according to standard procedures by two expert uropathologists. Pathological tumor size, pathological tumor capsule, positive surgical margin (PSM), TNM stage and the Furman nuclear grade were checked (2, 16). Postoperative complications including hematuria, bleeding, blood transfusion, urine leakage and intestinal obstruction were observed. The postoperative hospital stay was recorded.

#### Follow-up

Follow-up was scheduled 4 times annually for the first and second year, 2 times annually for the 3^rd^ to 5^th^ year, and annually thereafter. Follow-up contents included history collection, physical examination, chest X ray, abdominal color Doppler ultrasound or CT alternately and eGFR based on the blood test of kidney function (17). The eGFR of each patient 6 months after surgery was compared with that before surgery.

### Statistical analysis

Independent-sample two-sided t-tests were used for the comparisons of mean attenuation values of tumor and normal renal cortex at 4 various stages. Wilcoxon signed-rank test was used to compare eGFR data variables between preoperative and postoperative. Statistical significance was set at p<0.05. The data were analyzed with the SPSS software, version 23.0.

## RESULTS

### 

#### Patient's characteristics

The descriptive characteristics of all the patients are shown in Table-1, 36 patients who underwent laparoscopic SE of localized chRCC between November 2010 and December 2016 at our institute were consecutively selected. There were 18 males and 18 females with a mean age of 50.1±13.0 years. 17 renal masses were on the left side and 19 renal masses were on the right side. Mean tumor diameter was 3.9±1.0 cm. Mean PADUA score was 8.0±0.9. The mean preoperative eGFR was 78.5±22.4 mL/min.

**Table 1:** Demographic Characteristics.

Characteristics		Value
N		36
Age (years) mean±SD		50.1±13.0
**Gender**		
	Male (no.)	18
	Female (no.)	18
BMI (kg/m^2^) mean±SD		23.8±3.6
**Location**		
	Left (no.)	17
	Right (no.)	19
Tumor diameter (cm) mean±SD		3.9±1.0
PADUA score mean±SD		8.0±0.9
Preoperative eGFR (mL/min) mean±SD		78.5±22.4

#### CT imaging analysis

**Figure 1:**
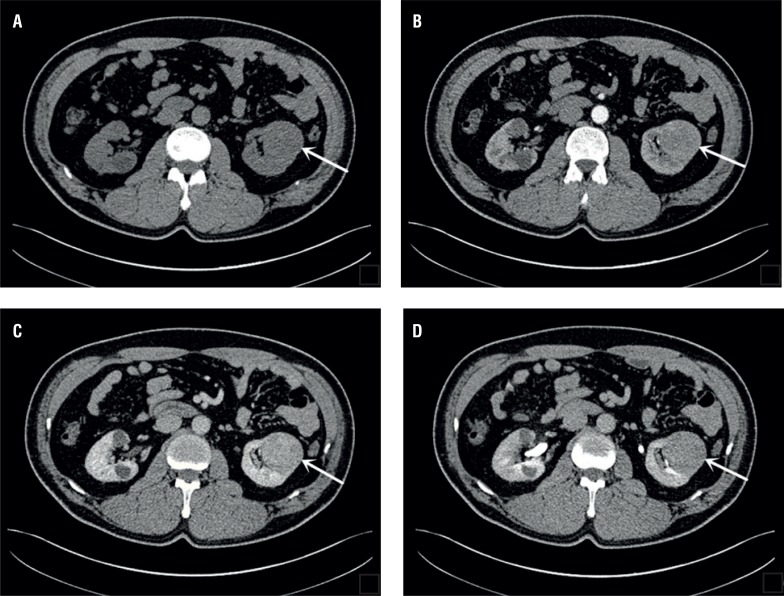
(A-D) Lesion is well-circumscribed and homogeneous on unenhanced (A), arterial (B), venous (c), and delayed (D) images, respectively.

All the tumors were solid masses with complete and inerratic pseudocapsule (Figures 1 A-D), 80% of tumors were homogeneous. Calcifications were not seen in any of these masses. None of the patients had evidence of lymph node or distant metastatic disease, either on initial staging CT or color Doppler ultrasound. Table-2 summarizes the attenuation values for the 36 patient's tumors over the unenhanced, arterial, venous, and delayed phases. The mean attenuation values of chRCC over the unenhanced, arterial, venous, and delayed phases were 39.2±4.3 HU, 75.7±8.1 HU, 97.1±10.2 HU, 74.3±7.5 HU respectively. The mean attenuation values of normal renal cortex over the unenhanced, arterial, venous, and delayed phases were 40.7±4.8 HU, 169.7±8.6 HU, 203.3±10.6 HU, 124.7±9.4 HU respectively. The attenuation values of chRCC were slightly lower than normal renal cortex at the unenhanced phase (P>0.05). The attenuation values of chRCC were significantly lower than normal renal cortex over the arterial, venous, and delayed phases (P<0.05).

**Table 2:** Mean attenuation values (HU) of tumor and normal renal cortex at 4 various stages.

Stage	Unenhanced	Arterial	Venous	Delayed
ChRCC	39.2±4.3	75.7±8.1	97.1±10.2	74.3±7.5
Renal cortex	40.7±4.8	169.7±8.6	203.3±10.6	124.7±9.4
t	1.40	47.74	43.32	25.15
P	0.17	<0.05	<0.05	<0.05

#### Operative and oncological data

The operative and oncological data are described in Table-3. Mean operative time and warm ischemia time were 104.3±18.2 min and 21.3±3.5 min, respectively, with a mean blood loss of 78.6±25.4 mL during the surgery. No patient revealed positive surgical margin pathologically. All tumors had a complete pathology pseudocapsule, 15 of the pseudocapsules had cancer cell infiltration, but none of the pseudo-capsules had cancer cell penetration (Figure-2 A and Figure-2 B). The arrows indicated the tumor complete pseudocapsule in Figure-2 A. In Figure-2 B, the arrows indicate the inner and outer parts of the tumor pseudocapsule with cancer cell invasion but without cancer cell penetration. The postoperative data are described in Table-4. The mean postoperative hospital stay was 5.3±1.5 days. Hematuria occurred in 3 patients and all disappeared within 3 days. No bleeding, blood transfusion, urine leakage or intestinal obstruction occurred. After a mean follow-up of 32.1±20.6 months, no patient had a local recurrence or metastatic progression. Postoperative eGFR of each patient was shown in Table-1. Mean eGFR was 70.5±19.2 mL/min. Postoperative eGFR was slightly lower compared to preoperative value with Wilcoxon signed-rank test. There was no significant difference between the two sets of data (P>0.05).

**Figure 2 A:**
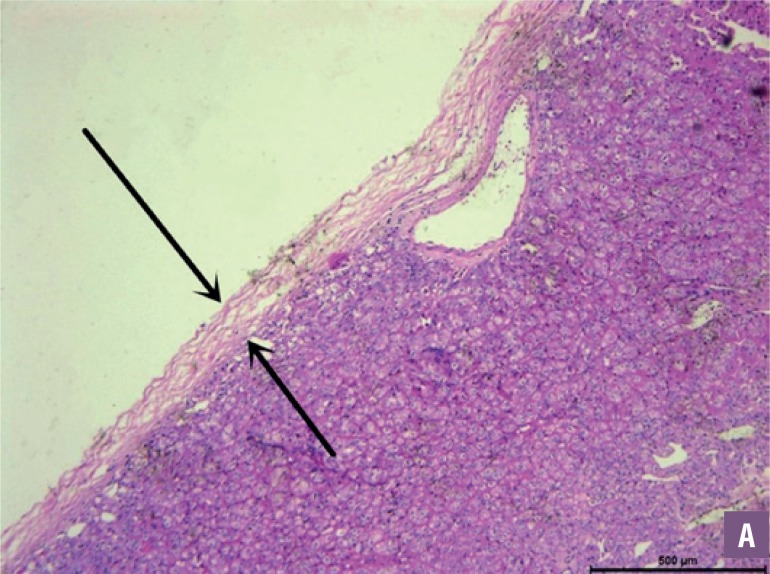
Microscopic investigation reveals chRcc with intact pseudocapsule (HE*40).

**Figure 2 B:**
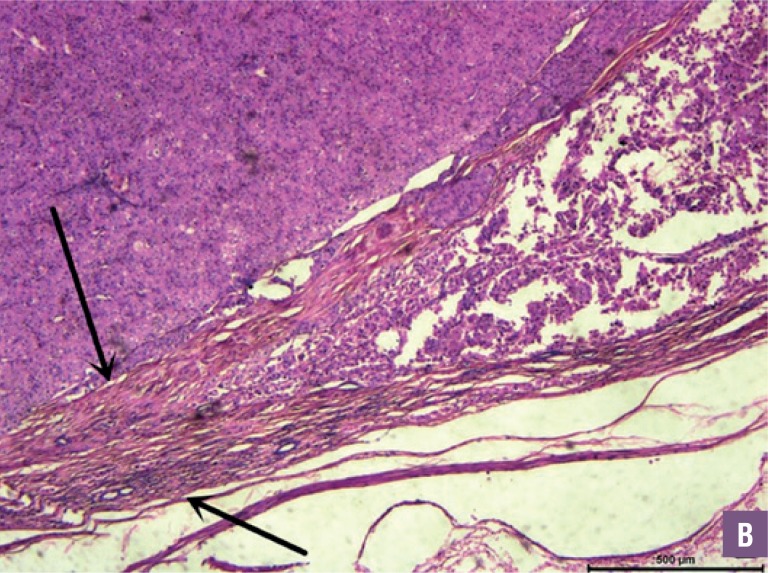
Microscopic investigation reveals the pseudocapsule of chRcc had cancer cell infiltration, but without penetration (HE*40).

**Table 3:** Operative and Oncological Data.

Characteristics	Value
N	36
Operation time (min) mean±SD	104.3±18.2
Warm ischemia time (min) mean±SD	21.3±3.5
Blood loss (mL) mean±SD	78.6±25.4
Positive surgical margin (no.)	0
Complete pseudocapsule (no.)	36
Pseudocapsules with cancer cell infiltration (no.)	15

**Table 4:** Postoperative Data.

Characteristics	Value
Postoperative hospital stays (days)	5.3±1.5
mean±SD	
Complication (no.) (%)	3(8.3%)
Hematuria (no.)	3
Bleeding (no.)	0
Blood transfusion (no.)	0
Urine leakage (no.)	0
Intestinal obstruction (no.)	0
Follow-up (months) mean±SD	32.1±20.6
Patients with recurrence (no.)	0
Postoperative eGFR (mL/min) mean±SD	70.5±19.2

## DISCUSSION

ChRCCs are the third most common sub-type of RCC and account for 3% - 5% of RCCs (1). Lipworth L, et al. (18) reported that the incidence of chRCC was significantly higher in the female population. In the series, the mean age of the patients was 50.1±13.0 and 50% of the patient population were women. Compared with one of the largest cohort recently (19), which included 166 chRCC patients, where the mean age was 48.5±12.9 and the female population was 51.4%, the results were similar. However, no previous study has demonstrated the etiology of age and gender difference of chRCC.

It has been evidenced that multiphasic CT is useful for distinguishing the benign vs. malignant renal masses and discriminating the pathological subtypes of RCC (20, 21). In the study, chRCC tends to be homogenous, solid, well circumcised and without calcification. Braunagel M, et al. (21) compared the CT imaging which features different subtypes of renal cell carcinoma (RCC), it was concluded that chRCCs were the most homogeneous tumors with fibrous strands and microbleeding without detection of hyalinization or necrosis. According to Zhang J's study (22), heterogeneous renal lesions were more likely to behaving aggressively. Thus, based on the relatively nonaggressive biologic behavior of chRCCs (4), it is no wonder that chRCCs are more likely to have homogenous appearance. The differentiation of the histology of renal tumors using multiphasic CT is based on the tumor blood supply. Previous study revealed that clear cell RCCs and oncocytomas tended to be hypervascular, chromophobe lesions and angiomyolipomas tended to enhance moderately, and papillary lesions were mostly hypovascular (22). In the study, chRCC appeared to be significantly hypoenhancing compared with normal renal cortex. The mean attenuation values of the chRCC was 75.7±8.1 HU, 97.1±10.2 HU, and 74.3±7.5 HU in the arterial, venous, and delayed phases, respectively. These values were comparable to those from a study by Wu J, et al. (23) who reported attenuation values of 63.2±5.2 HU, 89.8±11.1 and 64.8±7.2 HU in the corticomedullary, nephrographic, and excretory phases, respectively.

The treatments of localized chRCC include active surveillance (AS), radiofrequency ablation (RAF), radical nephrectomy (RN) and nephron-sparing surgery (NSS). Richard PO, et al. (24) conducted a study which included 81 oncocytoma and 14 chRCC diagnosed on biopsy and concluded that AS for renal neoplasms with oncocytic features is safe. However, as far as the authors are concerned, chRCC has a malignant biological behavior, AS may give patients great psychological burden. Hence, it is submitted that, AS may be a good treatment method for oncocytoma but not for chRCC. For the sake of minimally invasive therapy and maximally preserved renal function, several doctors will choose radiofrequency ablation (RFA) for treating RCC. Previous study showed that patients with T1b non-clear RCC (papillary RCC and chRCC) treated with percutaneous radio-frequency ablation provided comparable oncologic outcomes to partial nephrectomy (25). However, the accuracy of biopsy was not 100% and hybrid tumors have been classically described (26). In addition, as far as we are concerned, tumor size and location may be risk factors of oncological results in the patients who underwent radiofrequency ablation.

Surgical excision still remains the gold standard for the treatment of patients with renal masses including localized chRCC. Radical nephrectomy was once widely used to treat renal neoplasms because of its relatively simple performance, few complications and satisfactory oncological results. However, more and more studies showed that NSS had equivalent oncological outcomes to RN in treating T1a and T1b renal tumors (27, 28). Compare to RN, NSS preserves more normal renal parenchyma, which can reduce the potential risk of progressing chronic kidney disease. Bigot P, et al. (29) conducted a multicenter study including 234 patients with localized chRCC treated by NSS and concluded that oncological results of NSS for localized chRCC were excellent. NSS includes sharp excision, namely partial nephrectomy (PN) and blunt dissection, namely simple enucleation (SE). Given the advantages that SE had comparable oncological outcomes with PN and less complications than PN, SE was recommended to treat localized renal tumors (9–12). Even for some highly complex renal tumors, Serni S, et al. (8) reported that SE was an effective treatment with a potential key role to widen the NSS indications according to guidelines. In the current study, hematuria occurred in 3 patients and all disappeared within 3 days. No bleeding, blood transfusion, urine leakage or intestinal obstruction occurred. After a mean follow-up of (32.1±20.6) months, no patient had local recurrence or metastatic progression. SE appears to have controlled postoperative complications and excellent oncological results in treating localized chRCCs.

The key point of laparoscopic NSS is how to minimize WIT and maximize preservation of normal parenchymal. Compared to PN, SE has some advantages to minimize WIT. Mukkamala A, et al. (11) compared the WIT between SE and PN and found that the mean ischemia and operative times were 4 and 32 minutes shorter in the enucleation group, respectively. Perhaps because the renal sinus was less frequently entered and tumor bed suturing was less frequently needed, WIT was saved in SE group. Furthermore, SE can be conducted without hilar clamping more often than PN (12). In the study, mean operative time and warm ischemia time were 104.3±18.2 min and 21.3±3.5 min, respectively. The results are comparable to previous studies (11, 12). SE is possible because most localized renal masses are enveloped by a peritumoral pseudocapsule containing muscle, reticulin and collagens (6). According to Wang L's study (6), chRCC had the highest rate of extra-pseudocapsular extension and the highest percentage of tumors with larger (≥0.2 mm) intra-pseudocapsular arteries. In the current study, all the tumors were well enveloped by pseudocapsular. While SE can maximize preservation of normal parenchymal, PSM is a problem that cannot be ignored. The previous Lu Wang's study reported that the PSM rate was significantly higher in SE group than PN group and the local recurrence of SE group was comparable to PN group (30). However, even though PSM never evolves into a clinically significantly recurrence, rigorous postoperative surveillance may lead to emotional and financial burdens on the patients. Therefore, as far as the authors are concerned, SE should be performed for the selective patients with well circumcised renal masses. As a result, no PSM or local recurrence occurred in the study.

There are some limitations to this study. First, the study is limited by a relatively small sample because of the rarity of chRCCs. Second, the mean follow-up of 32.1±20.6 months might not be long enough to detect the long-term oncologic outcomes. This is partly because the concept of SE has been accepted by the urologists and SE has been performed for chRCCs at our institute since 2010. Third, there are some bias in the use of retrospective analysis, although data were carefully collected in a prospectively maintained database. Despite these limitations, the authors understand, that the current study is the first study to evaluate the preoperative imaging manifestation and therapeutic effect of laparoscopic simple enucleation (SE) for localized chRCC.

## CONCLUSIONS

Localized chRCCs have a great propensity for homogeneity and complete pseudocapsule. The attenuation values were slightly lower than normal renal cortex and small degree of enhancement. Based on the homogenous appearance and well circumcised image manifestation, laparoscopic SE is a safe and effective treatment for localized chRCC with little effect on renal function. The follow-up oncological results were satisfactory.

## COMPLIANCE WITH ETHICAL STANDARDS

All procedures performed in studies involving human participants were in accordance with the ethical standards of the institutional research committee and with the 1964 Helsinki Declaration and its later amendments or comparable ethical standards.

Informed consent Informed consent was obtained from all individual participants included in the study.

## Supplemental table Characteristics of the patient population.

**Table:**

Patient	Age (years)	Gender (M/F)	Location	Size(cm)	PADUA score	Preoperative eGFR(mL/ min)	Postoperative eGFR(mL/ min)
1	21	M	Left	2.5*2.0*2.0	9	139.0	129.0
2	57	M	Left	3.0*2.5*2.0	8	97.6	86.6
3	38	M	Right	4.0*3.2*2.5	9	91.3	76.2
4	24	F	Right	2.5*2.3*1.5	8	118.5	90.9
5	44	M	Right	5.0*4.8*3.0	8	100.7	89.2
6	55	M	Left	4.8*3.5*1.5	9	96.4	87.9
7	57	F	Left	2.0*2.0*1.5	7	82.2	73.3
8	51	F	Left	3.8*3.5*2.5	6	65.0	53.3
9	68	F	Right	6.0*4.5*3.0	7	62.9	74.3
10	51	F	Right	5.0*4.3*4.0	8	77.5	67.8
11	46	F	Left	3.0*3.0*2.5	7	77.9	80.4
12	57	F	Left	4.2*3.8*3.2	8	26.7	37.9
13	67	F	Right	4.0*3.4*3.0	7	66.9	52.2
14	49	M	Left	5.1*4.9*4.3	9	70.8	56.6
15	40	F	Right	3.0*2.5*2.0	8	74.3	104.1
16	42	M	Right	3.0*2.0*2.0	7	70.6	60.1
17	71	M	Left	4.8*4.5*4.0	8	70.3	64.9
18	41	F	Right	3.5*2.5*2.0	7	120.9	99.1
19	36	F	Left	4.7*4.4*4.0	9	84.5	70.7
20	54	F	Right	4.3*3.8*3.5	8	96	84.6
21	59	F	Right	4.5*3.5*3.5	7	64	61.4
22	58	M	Right	4.5*4.3*4.0	8	76.1	64.7
23	27	M	Right	5.0*4.0*3.5	9	89.9	76.4
24	56	M	Right	4.8*5.1*5.0	10	70.4	57.7
25	48	M	Left	4.5*4.2*3.5	9	71.1	57.4
26	72	M	Left	4.1*4.2*2.5	9	67.7	62.0
27	52	F	Left	3.1*2.5*2.1	7	91.5	74.8
28	58	M	Right	2.5*2.2*2.2	8	78.9	84.2
29	34	F	Left	5.0*5.0*4.5	8	103.5	71.8
30	64	M	Right	2.8*2.6*2.5	9	64.8	69
31	34	M	Right	2.0*1.6*0.8	6	25.4	23.7
32	51	M	Left	3.6*3.0*2.5	7	59.4	66.5
33	48	M	Right	4.5*4.0*4.4	8	108.2	117.3
34	44	F	Left	3.8*3.3*3.5	8	77.4	65.2
35	56	F	Right	5.0*5.0*4.5	9	62.9	53.7
36	72	F	Left	3.5*4.0*3.0	8	66.0	48.6
